# Synthesis and Mechanism Study of an Environmental Additive Used in Water-Based Drilling Fluids from Bovine Bone Glue

**DOI:** 10.3390/ma17225547

**Published:** 2024-11-13

**Authors:** Lei Guo, Jie Yang, Lubin Yu, Bingqian Song, Weichao Du

**Affiliations:** 1 Oilfield Chemicals R&D Institute, China Oilfield Services Co., Ltd., Yanjiao 065201, China; guolei5@cosl.com.cn (L.G.); yangjie46@cosl.com.cn (J.Y.); yulb3@cosl.com.cn (L.Y.); 2 Shaanxi Province Key Laboratory of Environmental Pollution Control and Reservoir Protection Technology of Oilfields, Xi’an Shiyou University, Xi’an 710065, China; 18066702698@163.com

**Keywords:** bovine bone glue, epichlorohydrin, water-based drilling fluids, clay swelling, inhibition

## Abstract

At present, animal bone glue has been widely used in industry, but there are no relevant research reports on its application in the petroleum industry. In this paper, the rheological properties, inhibition, filtration, and temperature resistance performance of modified bone glue (Mbg) were evaluated in water-based drilling fluids, and the results showed that Mbg can significantly affect the performance of water-based muds with minimal dosage, and temperature resistance of Mbg could reach up to 130 °C. The inhibition mechanism of Mbg in drilling fluids was investigated by infrared spectroscopy (FT-IR), X-ray diffraction (XRD), zeta potential, thermogravimetric analysis (TGA), and scanning electron microscope (SEM). Results revealed that when 2% Mbg was added, a three-dimensional network structure was formed in an aqueous solution, which reduced the water content from 4.83% to 4.23%. FT-IR analysis showed that Mbg strongly adsorbed onto clay through hydrogen bonding, which reduced the clay particles in based muds from 1.251 µm to 0.789 µm and effectively controlled the filtration loss of the drilling fluids.

## 1. Introduction

Drilling fluids play a crucial role in ensuring fast, safe, and efficient drilling. In recent years, with the implementation of new environmental laws, people’s environmental awareness has gradually increased, and higher and updated requirements have been put forward for drilling processes. Scientific researchers are paying more and more attention to the impact and damage of drilling fluids on the surrounding environment during drilling [1,2].

There are various components in drilling fluids, including salt, heavy metals, polymers, asphalt, etc. If they are not treated and released in a timely manner, it will lead to a series of issues such as land salinization and heavy metal pollution [3,4,5,6]. In environmentally sensitive areas, oil-based drilling fluids, water-based drilling fluids containing sulfonated treatment agents, and waste liquids are listed in the Chinese National Hazardous Waste List and cannot be used.

Currently, commonly used non-sulfonated CMC/PACs have a temperature resistance of less than 120 °C, modified starch additives have a temperature resistance of less than 150 °C, and polyacrylamide derivatives temperature resistance exceeds 200 °C [5]. However, such drilling fluid additives are expensive and have poor biodegradability, and in certain situations when drilling in areas with high environmental requirements, sometimes suitable drilling fluid treatment agents are not available, which poses a serious challenge to the design and on-site construction of deep drilling fluid. Therefore, the development of environmentally friendly drilling fluid systems has become increasingly important to the oil field industry [5,6].

For the past few years, researchers around the world have investigated the application of natural materials in water-based drilling fluids and obtained significant achievements [7,8]. Al-Hameedi et al. [9] researched banana peel powder (BPP), which was added in various amounts of water-based drilling fluids. The results indicated that when the BPP concentration was 3%, the rheological properties of the drilling fluid were effectively increased. Yalman et al. [10] studied the performance of rice husk ash when added to drilling fluids, the results showed that the filtration loss of drilling fluid was reduced by 10% when 4% of rice husk ash was added, and the apparent viscosity of the drilling fluid could be increased by 60% when the concentration of the rice husk ash was 15%. However, the existing drilling fluid additives have shortcomings such as temperature resistance and environmental protection, which still cannot meet current complex drilling needs, so it is of great significance to develop environmentally friendly and temperature-resistant drilling fluids [11,12,13].

As a green and biodegradable natural polymer compound, animal bone glue has been used for more than 3000 years and has broad development prospects [14,15]. Animal bone glue consists of animal tissues as raw materials extracted through industrial processing of animal natural adhesives and has been widely used as adhesives and in other fields [16,17]. For example, Dušek et al. [18] investigated a composite building material based on rape straw and an environmentally friendly adhesive (sodium lignosulfonate bone glue). The results indicated that bone glue combined with sodium lignosulfonate was found to be suitable to produce natural binders in insulation materials based on rape straw. Hou et al. [19] synthesized osteogenic double cross-linked biodegradable bone glue with enhanced mechanical properties for fracture fragments. At present, animal bone glue and its modified materials have wide and large-scale applications in various fields such as medicine, food, construction, rubber, cosmetics, etc. [20,21,22,23], but, so far, there have been no literature reports on the application of bone glue in water-based drilling fluids.

Based on the above analysis, in order to provide more choices for environmentally friendly drilling fluids, expand the application fields of bone glue, and continuously improve the temperature resistance of environmentally friendly materials, in this paper, we have developed a modified bone glue (Mbg) using bovine bone glue as the raw material, epichlorohydrin as the modifier, and sodium hydroxide as the alkaline hydrolysis agent, and evaluated its performance in drilling fluid.

## 2. Experimental

### 2.1. Materials

Sodium hydroxide was obtained from Tianjin Tianli Chemical Reagent Co., Ltd., Tianjin, China, epichlorohydrin was supplied by Tianjin Komeo Chemical Reagent Co., Ltd., Tianjin, China, and anhydrous ethanol was procured from Tianjin Fuyu Fine Chemical Co., Ltd., Tianjin, China, all of which were analytically pure. Bovine bone glue was collected from Henan Kyle Chemical Products Co., Anyang, China, Sodium montmorillonite (Na-MMT) with cation exchange capacity 82 meq/100 g (0.82 mmol/g) was obtained from Xiazijie Bentonite Technology Co., Ltd., Xinjiang, China, and its purity was 95%.

### 2.2. Synthesis of Mbg

**Figure 1:**
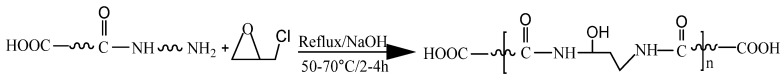
Synthetic route of Mbg.

**Figure 2:**
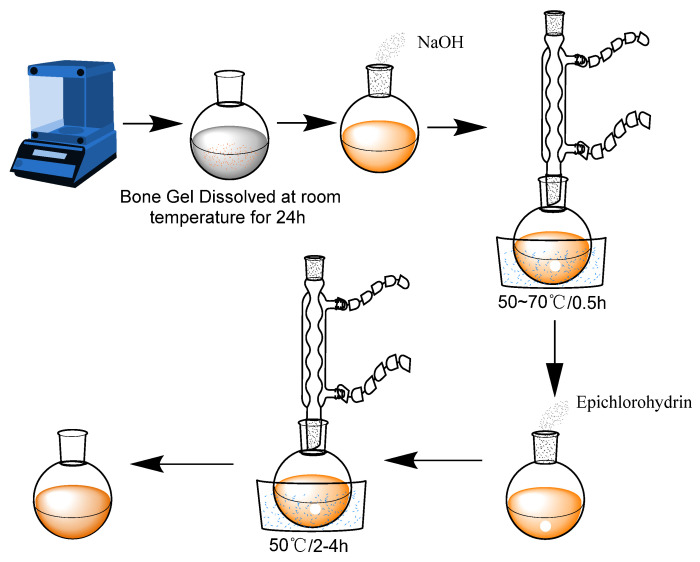
Process diagram for the preparation of modified bone glue.

At room temperature, bone glue was fully swollen in distilled water for 24 h, and 3.6 g bone glue solution, 25 g distilled water, and 1.5 g NaOH was added into a round bottomed flask. The flask was quickly heated to 50~70 °C and stirred for 30 min. Then, 0.5 g epichlorohydrin was added and further reacted for 2 h. Substance was removed from the flask and Mbg was obtained. Figure 1 shows the synthetic route of Mbg and the process diagram for the preparation of modified bone glue is shown in Figure 2.

### 2.3. Characterization

A mixture of modified bone glue and potassium bromide in a certain proportion were ground together for FT-IR analysis by Thermo Fisher Nicolet iS5 Fourier Transform Infrared Spectrometer (Anton Paar, Graz, Austria) at the wave number range of 40,500–400 cm^−1^. For SEM, the samples were prepared in accordance with the method described in Section 2.1, the microstructure of Mbg was analyzed by scanning electron microscopy (SEM) using FEI Quanta 450 scanning electron microscope (Electron, Tokyo, Japan).

### 2.4. Performance Evaluation Experiments of Mbg

#### 2.4.1. Drilling Fluids Performance Evaluations

Filtration refers to the phenomenon of free water in drilling fluids penetrating the pores of wellbore rock under the action of pressure difference, which is called the filtration effect of drilling fluids. Usually, we use filtration loss or water loss to indicate the quality of filtration. The effect of Mbg on filtration loss of drilling fluids was studied by using a quadruple medium-pressure filtration loss meter (Qingdao Tongchun Petroleum Instrument Co., Ltd., Qingdao, China) at a pressure of 0.69 MPa.

The rheology is one of the most fundamental properties of drilling fluids, which refers to the characteristic of flow deformation of drilling fluid under external forces. This characteristic is usually described by rheological parameters such as apparent viscosity (AV), plastic viscosity (PV), and dynamic shear force (YP). High-performing drilling fluids can greatly reduce drilling costs and improve drilling efficiency. Rheological properties of basic muds with/without Mbg were tested using a six-speed rotational viscometer (Qingdao Tongchun Petroleum Instrument Co., Ltd., China), and rheological parameters were calculated by the following equations:

(1) $$\mathrm{AV}=(\frac{1}{2})\varphi 600$$

(2) PV=φ600−φ300

(3) YP=(φ300−PV)

where AV is apparent viscosity (mPa·s), PV is plastic viscosity (mPa·s), and YP is yield point (Pa).

#### 2.4.2. Linear Swelling Experiments

A total of 10 g 100 mesh bentonite powder dried at 105 °C was placed into a measuring bucket, its depth L_1_ was measured with a vernier caliper, and the bucket was compacted under a pressure of 10 MPa for 5 min to obtain an artificial rock core with a depth of L_2_. The linear expansion rate of artificial rock cores on a linear swelling meter was measured, and the initial readings R_1_ and the readings R_2_ and R_3_ at 2 h and 16 h, respectively, were recorded. The linear swelling rate was calculated according to Formula (4).

(4) $$S=\frac{R_{3}-R_{1}}{L_{1}-L_{2}}\times 100\%$$

where S is linear swelling rate, %.

### 2.5. Inhibition Mechanism Analysis of Mbg

#### 2.5.1. FT-IR Analysis

Na-MMT was subjected to FT-IR analysis according to the method described in Section 2.1. Na-MMT treated with 2% Mbg was centrifuged and filtered in a vacuum pump, during the filtration process, and Mbg/Na-MMT was carefully rinsed 4–5 times with anhydrous ethanol/ethyl acetate. Then, Mbg/Na-MMT was dried at 105 ± 1 °C for 24 h and stored in a sealed container for future testing. The samples were mixed with potassium bromide in a certain proportion and ground together for FT-IR analysis by Thermo Fisher Nicolet iS5 Fourier Transform Infrared Spectrometer at the wave number range of 40,500–400 cm^−1^.

#### 2.5.2. TGA

Samples preparation was according to the method described in Section 2.1, and determined by a thermogravimetric analyzer (Mettler Toledo, Greifensee, Switzerland). The nitrogen flow rate was set at 10 mL/min, and the heating rate was 20 °C/min.

#### 2.5.3. XRD Analysis

Samples preparation was according to the method described in Section 2.1, and analyzed used an X-ray Polycrystalline Diffractometer (Brook D8 ADVANCE, Karlsruhe, Germany). The scanning range and scanning speed was 5~80° and 10 °/min, respectively.

#### 2.5.4. Zeta Potential Analysis

Samples preparation was according to the method described in Section 2.1. The average particle size was determined using a Zeta Potential Analyzer (Brookhaven, NY, USA) of basic muds for 16 h of hot rolling before and after the addition of Mbg.

#### 2.5.5. SEM Analysis

Samples preparation was according to the method described in Section 2.1. The microstructure of Mbg was analyzed by scanning electron microscopy (SEM) using a FEI Quanta 450 scanning electron microscope (Electron, Japan).

## 3. Results and Discussion

### 3.1. Characterization

**Figure 3:**
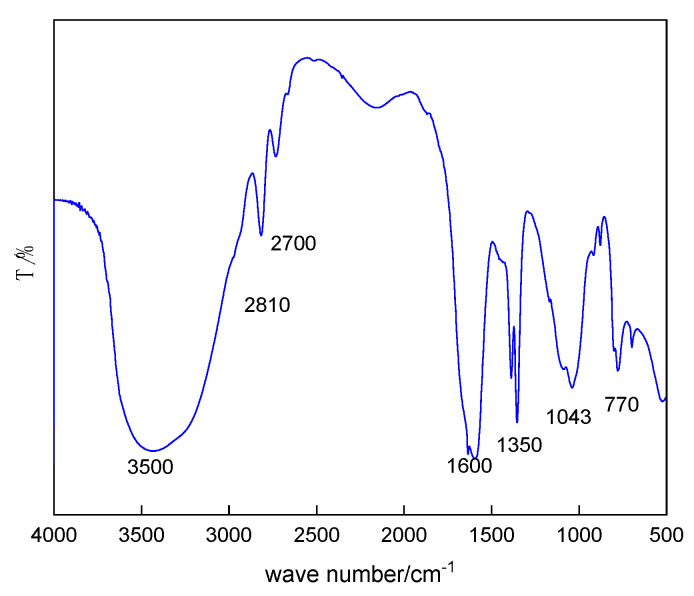
FT-IR of Mbg.

Mbg’s structure was determined by FT-IR, as shown in Figure 3.

As shown in Figure 3, the absorption peak at 3500 cm^−1^ is related to the hydrogen bond peak of O-H and N-H, and characterized as a stretched oscillation peak of the amino group. The bending vibration that appeared at 1600 cm^−1^ indicated that after the reaction between the bone glue and epichlorohydrin, the peptide bonds on the molecular chain of the animal bone glue were still retained. The absorption peaks at 2810 cm^−1^, 2700 cm^−1^, and 1350 cm^−1^ were attributed to O-H stretching vibration, and the peaks at 1043 cm^−1^ and 770 cm^−1^ were shown to be bending vibration peaks and proved that the C-H and ether groups were present. All the results signified that the epoxy bond of epichlorohydrin molecules was successfully broken after grafting into the molecular chain of animal bone glue, and Mbg was synthesized successfully.

### 3.2. Performance Evaluation Experiments

**Figure 4:**
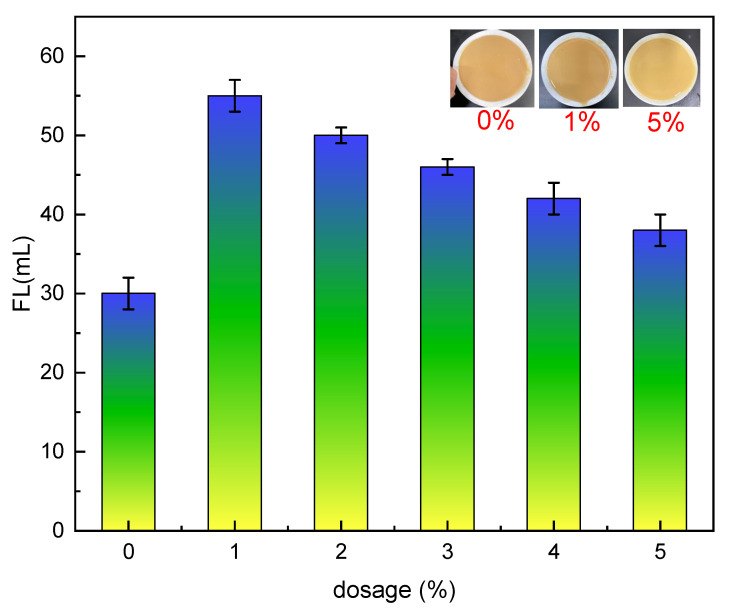
The effect of Mbg concentration on the filtration loss of basic muds.

**Figure 5:**
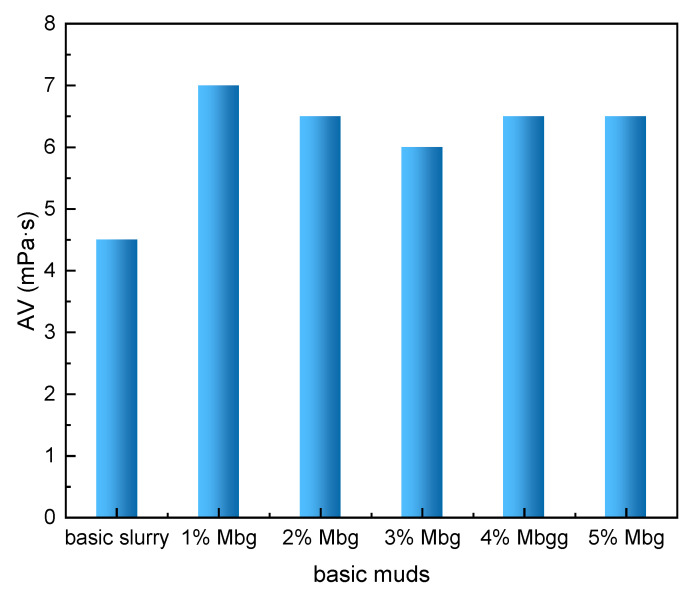
The effect of Mbg concentration on the apparent viscosity of basic muds.

The effect of 1–5% Mbg on the filtration loss reduction performance, rheological properties of drilling fluids, and linear swelling experiments were investigated before and after hot rolling at 120 °C for 16 h. The results are displayed in Figure 4 and Figure 5 and Table 1.

As shown in Figure 4, the basic mud filtration loss was 30 mL. When Mbg was increased, the filtration loss grew instead, and the findings demonstrated that Mbg was ineffective in lowering filtration loss. Reducing the drilling fluids filtration loss is the main function of the filtrate loss reducer, which participates in mud cake formation or reduces the liquid phase viscosity to achieve the effect of reducing filtration loss. When its dosage was less than 1%, the filtrate loss reducer did not completely block the mud cake channel, but when the dosage exceeds 1%, it plays an excellent role in reducing filtration.

**Figure 6:**
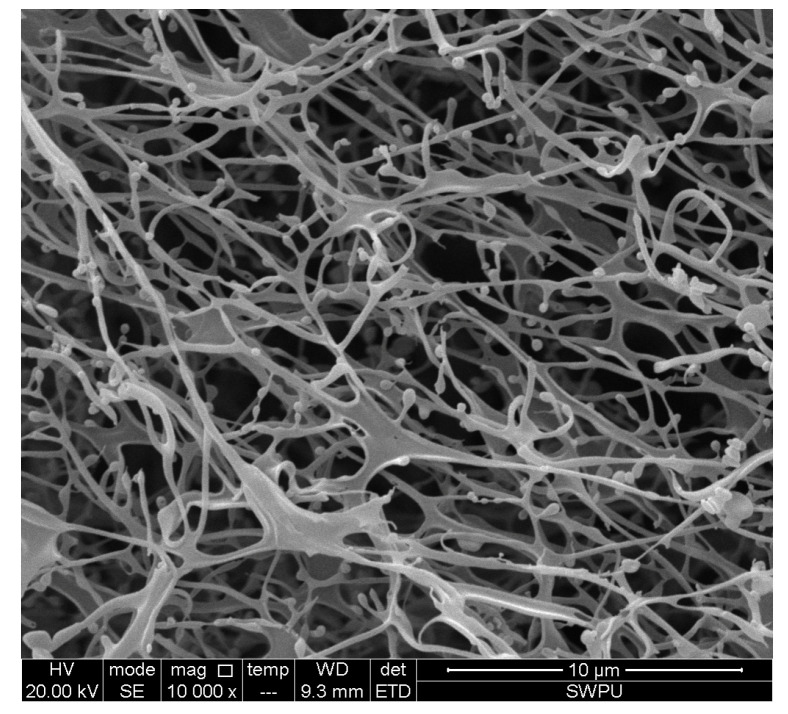
The SEM image of Mbg solutions.

As shown in Figure 5, when Mbg (1–5%) was added, the apparent viscosity of basic muds was raised. The reason why the viscosity of the basic muds increased was mainly due to the formation of a dense three-dimensional network structure in the MBG solutions, which was observed by SEM, Figure 6.

As shown in Figure 6, Mbg exhibited a three-dimensional spatial network structure with a relatively smooth and densely latticed surface. Moreover, some branched structures appeared in its microstructure, which were located on the surfaces of the pore between the lattices, and the results revealed that the stability of these branching structures with the addition of Mbg was improved [24].

**Figure 7:**
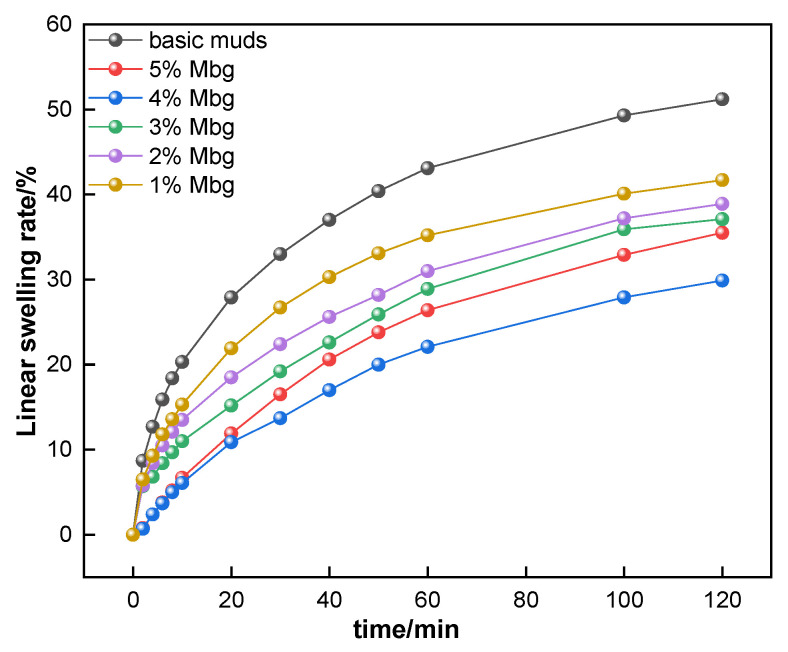
The effect of Mbg concentration on the linear swelling rate.

The inhibition performance of Mbg in basic muds was accessed. Results are presented in Figure 7.

As shown in Figure 7, the basic mud linear swelling rate can be reduced by adding Mbg, in which 2% Mbg could reduce the linear swelling rate of the basic muds from 50.2% to 29.9%. Analysis revealed that Mbg can be adsorbed onto the Na-MMT and promoted interlayer dehydration of clay crystals, thus, effectively inhibiting clay hydration [25].

**Figure 8:**
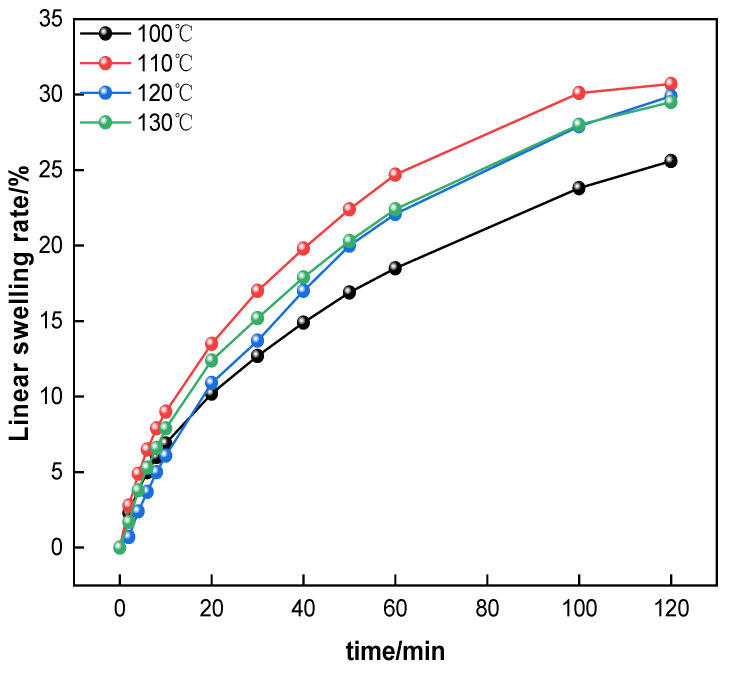
The effect of temperature on the linear swelling rate of 2% Mbg.

Based on the above results, 2% Mbg’s temperature resistance was studied at 100–130 °C. The results are illustrated in Figure 8.

According to Figure 8, when 2% Mbg was added and the action temperature was 100 °C, the basic muds’ linear swelling rate was 25.6%. When the temperature was 110 °C and 120 °C, the linear swelling rate was 30.7% and 29.9%, respectively. At 130 °C, the linear swelling rate was 29.5%. According to the above experimental results, Mbg’s temperature resistance could be attained at 130 °C.

### 3.3. Inhibition Mechanism Analysis

#### 3.3.1. FT-IR Analysis

**Figure 9:**
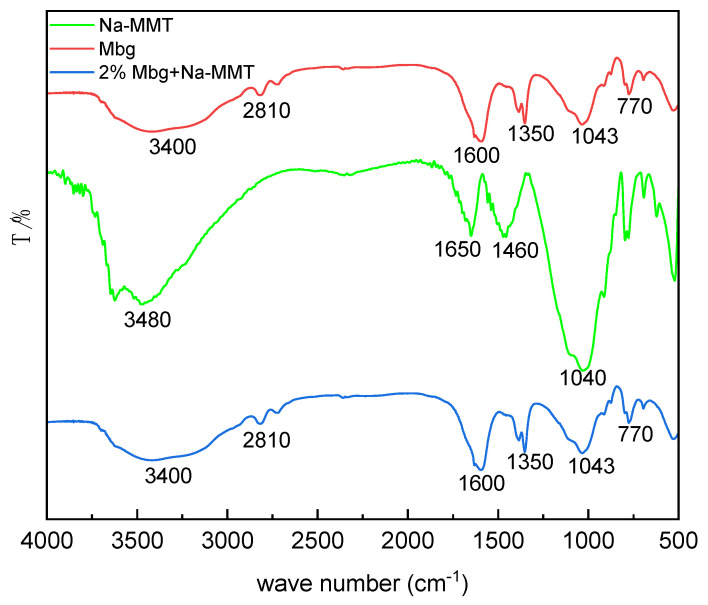
FT-IR spectra of Mbg, Na-MMT, and 2% Mbg/Na-MMT.

The structure was studied by FT-IR analysis of Mbg, Na-MMT, and 2% Mbg/Na-MMT, and the results are displayed in Figure 9.

As shown in Figure 9, the absorption peaks of Mbg/Na-MMT at 2810 cm^−1^ and 1350 cm^−1^ were attributed to O-H stretching vibration peak. The absorption peak at 3400 cm^−1^ contributed to the hydrogen bond between O-H and N-H, and considered to be the telescopic vibrational peak of the amino group. The bending vibration that appeared at 1600 cm^−1^ demonstrated that -NH_2_ was present on Mbg/Na-MMT. The bending vibration peaks at 1043 cm^−1^ and 770 cm^−1^ indicated that the C-H and ether groups had formed. All the results signified that after the addition of 2% Mbg, it was taken up by Na-MMT and the stability of the Na-MMT structure was improved.

#### 3.3.2. TGA

**Figure 10:**
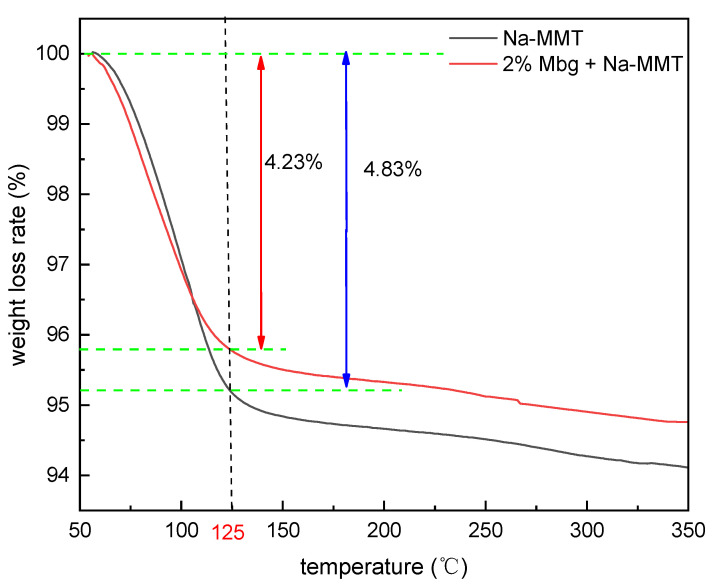
Thermogravimetric analysis comparison chart.

Mbg’s temperature resistance was analyzed by TGA. The results are displayed in Figure 10.

The mass loss within the temperature range of 50~125 °C was attributed to the volatilization of moisture in the Na-MMT. Furthermore, we found that the mass loss rate of pure Na-MMT was 4.83%. The volume loss rate of Na-MMT decreased to 4.23% after the addition of 2% Mbg, indicating that Mbg was adsorbed onto Na-MMT and replaced some of the absorbed water on its surface. In addition, the quality of Na-MMT and Na-MMT /Mbg continued to deteriorate at the temperature range of 125–350 ° C, which was due to the structural damage of Mbg at high temperatures.

**Figure 11:**
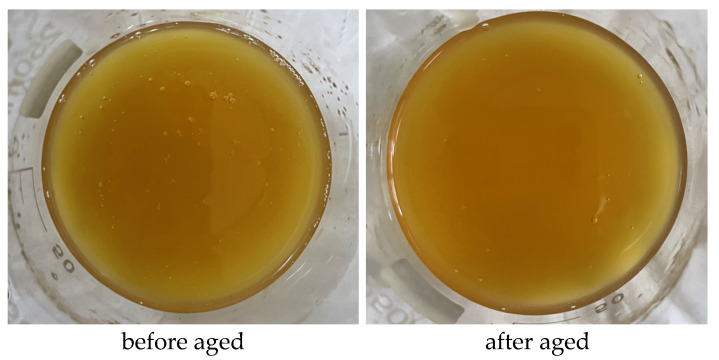
Comparison of Mbg before and after aging.

In order to intuitively study the dispersion state of Mbg at high temperatures, we placed 2% Mbg in a roller heating furnace at 120 ° C and rolled 2% Mbg for 16 h. As shown in Figure 11, the aging state of Mbg at high temperatures did not change significantly, indicating Mbg has a nice thermal stability at 120 ° C. The reason may be that Mbg has excellent temperature resistance, and its molecular structure is not destroyed at high temperatures.

#### 3.3.3. XRD Analysis

**Figure 12:**
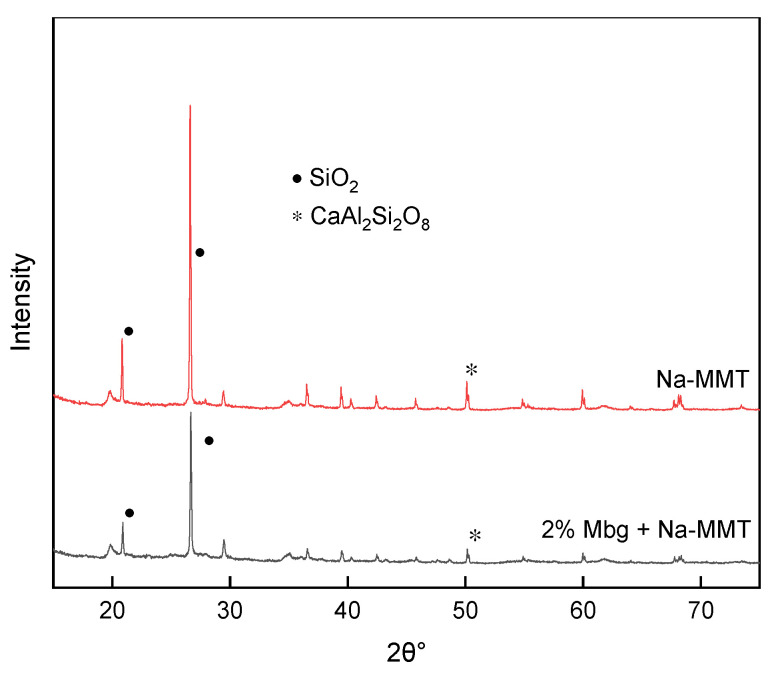
XRD analysis.

The ingredients of Na-MMT and 2% Mbg/Na-MMT were investigated by XRD analysis. The results are exhibited in Figure 12.

As shown in Figure 12, strong SiO_2_ peaks with diffraction peaks appeared at 21° and 27°, and a CaAl_2_Si_2_O_8_ characteristic peak was observed at 50°. In contrast to the basic muds’ formulation experiments, the characteristic peaks found in the XRD spectra are in general agreement consistent with the ingredients of recipients. Observation of the basic muds’ XRD spectra before and after the addition of 2% Mbg showed that there was a corresponding peak, but the peaks became smaller, which suggested indirectly that the primary functional groups of Mbg were absorbed in the clay, and the adsorption effect was clearly evident. Based on this, Mbg was successfully composited.

#### 3.3.4. Zeta Potential Analysis

**Figure 13:**
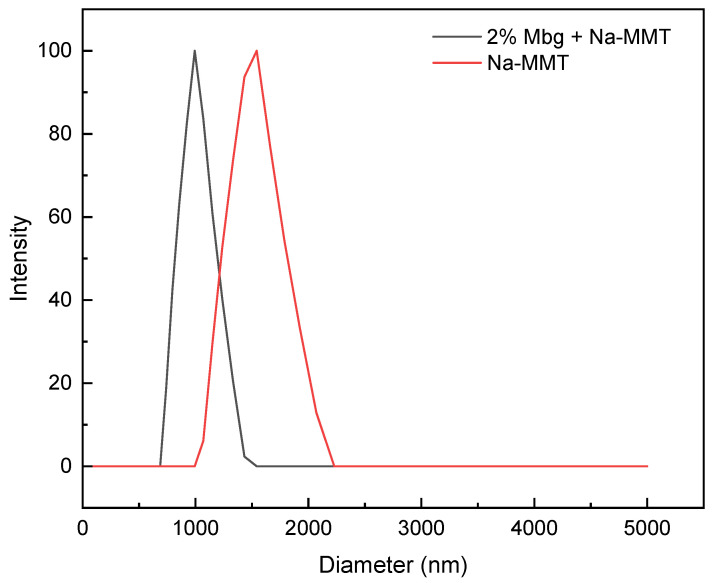
Grain size analysis comparison chart.

The average grain size were determined by zeta potential analysis of Na-MMT and 2% Mbg/Na-MMT. The results are presented in Figure 13.

As shown in Figure 13, Na-MMT’s average grain size was 1.251 µm, and after 2% Mbg was added, the average grain size of Na-MMT decreased to 0.789 µm. The results revealed that Na-MMT’s average grain size was decreased by Mbg, and Mbg’s nice dispersion and the agglomeration and precipitation was difficult to avoid.

**Figure 14:**
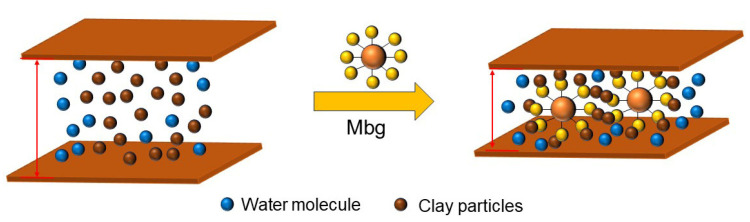
Inhibition mechanism of Mbg.

From the above experimental results and as shown in Figure 14, the action mechanism of Mbg involved a number of reactive functional groups present in its molecular structure, which were chemically cross-linked by functional groups like hydroxyl and amine. Therefore, the quantity of the molecular was increased and several new functional bonds were generated, these bonds were absorbed by the drilling fluids and displayed a nice inhibitory ability.

## 4. Conclusions

In this work, an eco-friendly water-based drilling fluids additive Mbg was prepared by using animal bone glue, epichlorohydrin as the raw material, and sodium hydroxide as the alkaline hydrolysis agent, and were studied for rheological properties, filtration loss, and inhibition properties.

(1) The results revealed that when 2% Mbg was added at 120 °C, the linear swelling rate decreased from 50.2% to 29.9%. The filtration loss of the basic slurry was increased by the filtration loss reducing experiment and showed a poor filtration loss reduction impact. When the Mbg concentration increased, its apparent viscosity and plastic viscosity increased. The temperature resistance of Mbg was investigated, and the results showed that the temperature resistance of Mbg was up to 130 °C. Based on this, the results indicated that Mbg has better temperature resistance and inhibition. The microstructure of Mbg was observed by SEM, and revealed that Mbg’s structure was stable.

(2) Based on the analysis of the inhibition mechanism of Mbg, the FT-IR and XRD patterns showed the effective synthesis of Mbg and the adsorption of its main functional groups in the drilling fluids. It can be seen from the relevant experimental data that the modified bone glue (Mbg) prepared in this study has good inhibition of shale hydration and clay expansion and has good application prospects.

## Figures and Tables

**Table 1:** The effect of Mbg concentration on the rheological properties of basic muds.

Mbg Concentration (%)	PV (mPa·s)	YP (Pa)
0	4.0	1.0
1	6.0	1.0
2	6.0	1.0
3	8.0	1.0
4	6.0	5.0
5	8.0	3.0

## Data Availability

The original contributions presented in the study are included in the article, further inquiries can be directed to the corresponding author.
